# Intraoperative Video Analysis of Pancreatic Stump and Stapler Closure-Induced Pancreatic Fistula in Laparoscopic Distal Pancreatectomy: A Retrospective Study

**DOI:** 10.7759/cureus.58959

**Published:** 2024-04-24

**Authors:** Hisamichi Yoshii, Hideki Izumi, Rika Fujino, Eiji Nomuraa, Masaya Mukai

**Affiliations:** 1 Department of Gastroenterology, Tokai University Hachioji Hospital, Hachioji, JPN

**Keywords:** stapler closure, retrospective study, postoperative pancreatic fistula, laparoscopic distal pancreatectomy, laparoscopy, celiac axis

## Abstract

Objectives

Pancreatic stump closure in laparoscopic distal pancreatectomy (Lap-DP) is commonly performed using an automatic stapler. Herein, the magnification effect of laparoscopy was used to observe the pancreatic stump and retrospectively investigate factors that may cause postoperative pancreatic fistula.

Methods

This is a single-center retrospective study. We selected 62 cases of Lap-DP performed between March 2016 and May 2022. We retrospectively analyzed 54 cases where pancreatic transection sites could be observed using an intraoperative video. Pancreatic transection was performed using the Powered ECHELON FLEX®+ GST® System (Ethicon, Somerville, USA). For quantitative studies, we investigated the factors that cause pancreatic fistula and other factors causing pancreatic fistula.

Results

Pancreatic parenchymal hemorrhage and injury occurred in 22.2% and 29.6% of cases, respectively. International Study Group of Pancreatic Surgery grade B/C pancreatic fistula was observed in 12 cases (22.2%). Univariate analysis of pancreatic (n = 12) and nonpancreatic (n = 42) fistula groups showed no significant differences in pancreatic thickness. The pancreatic fistula group had a significantly high incidence of the hard pancreas (p = .009), pancreatic parenchymal bleeding (p = .002), and pancreatic parenchymal damage (p < .001). Multivariate analysis revealed that pancreatic parenchymal damage was an independent cause of pancreatic fistula (hazard ratio, 81.4 (8.5-772.3), p < .001).

Conclusion

Pancreatic parenchymal damage due to compression during pancreatic stump closure using an automatic stapler in Lap-DP may cause pancreatic fistula.

## Introduction

Postoperative pancreatic fistula (POPF) is a serious complication of distal pancreatectomy (DP) that can lead to complications, such as postoperative bleeding, delayed gastric emptying, and intra-abdominal abscess, thereby causing prolonged hospital stays and increased readmission and mortality rates [1-4]. Laparoscopic DP (Lap-DP) has become a mainstream approach, and pancreatic stump closure is typically performed using an automatic stapler [5-8]. Reinforced staplers using polyglycolic acid (PGA) for pancreatic stump reinforcement have also been used. However, randomized controlled trials (RCTs) comparing reinforced and standard staplers have not reported any clear effectiveness [9-13]. Furthermore, other methods such as PGA mesh application to the pancreatic stump [14] and slow firing method with an automatic stapler [15] have been reported. Currently, the incidence of POPF following DP is 5.5%-35% [9-15], and it has been reported to be associated with events such as pancreatic thickness and bleeding at the pancreatic stump [13,16-19].

We aimed to investigate factors causing POPF based on the magnification effect of laparoscopy to observe stapler closure of the pancreatic transection edge in Lap-DP.

## Materials and methods

Patient selection

A total of 72 patients clinically diagnosed with tumors or cysts in the pancreatic body/tail who underwent DP at Tokai University Hachioji Hospital between March 2016 and May 2022 were selected. Overall, 62 patients underwent Lap-DP, and the pancreatic stump could be observed using an intraoperative video in 54 patients. This was a single-center retrospective study that included all cases treated at the hospital between 2016 and 2022. No inclusion or exclusion criteria were applied in this study.

Our facility generally attempts a laparoscopic approach for patients undergoing DP. For tumors with infiltration into the celiac or common hepatic artery, DP with celiac axis resection (n = 1) is performed via open surgery. Conversion from laparoscopy to open surgery occurred in two cases. Eight cases were excluded owing to the unavailability of video data or inadequate confirmation of the pancreatic stump. No surgery-related deaths within 90 days were noted. This study was conducted in accordance with the principles embodied in the 1975 Declaration of Helsinki, as revised in 2013, and was approved by the Tokai University Ethics Committee (approval number: 22R271; date of approval: March 28, 2023). The requirement for the acquisition of informed consent from patients was waived because of the retrospective nature of this study.

Patient data collection

We extracted and analyzed preoperative, intraoperative, and postoperative data from the hospital’s patient database. Preoperative data included the patient’s age, sex, body mass index (BMI), comorbidities, albumin level, glycated hemoglobin (HbA1c) level, and pancreatic thickness. Intraoperative and postoperative data included operative time, blood loss, pancreatic texture, pancreatic stump bleeding, pancreatic parenchymal bleeding, pancreatic parenchymal damage, amylase values on postoperative days 1 and 3, postoperative complications with Clavien-Dindo grade of ≥IIIa, duration of hospital stay, and readmission. Pathological data included tumor histology and size. We compared these factors between the pancreatic and nonpancreatic fistula groups using univariate analysis and conducted multivariate analysis to identify independent factors associated with pancreatic fistula.

Surgical technique and patient management

In Lap-DP, dissection starts from the inferior margin of the pancreas to identify the superior mesenteric vein. When tunneling through the pancreas, the pancreatic parenchyma was divided after clipping the splenic vessels. The pancreatic division was performed using Powered ECHELON FLEX 60 (Ethicon, Somerville, USA) with either a green (4.1 mm) or black (4.2 mm) cartridge without performing the slow firing method or gradual compression. Postoperatively, a negative-pressure silicone drain was placed in the abdominal cavity of all patients, and amylase levels were measured on postoperative days 1 and 3. No prophylactic antibiotics or somatostatin analogs were administered. On postoperative day 3, drain removal was performed based on the appearance of the drain fluid and amylase level (there were no clear criteria for amylase levels). If the drain fluid was wine-red in color or had a positive pus stain, it was assumed to indicate the presence of a pancreatic fistula. In such cases, the drain was kept in place and the fluid was replaced. The drain was replaced under x-ray with a guidewire due to the amount of pancreatic leakage and pus. The drain was removed when the amount of drainage was minimal. If an intra-abdominal abscess was detected following drain removal, drainage was performed under ultrasound or computed tomography (CT) guidance.

Preoperative CT and video data

**Figure 1:**
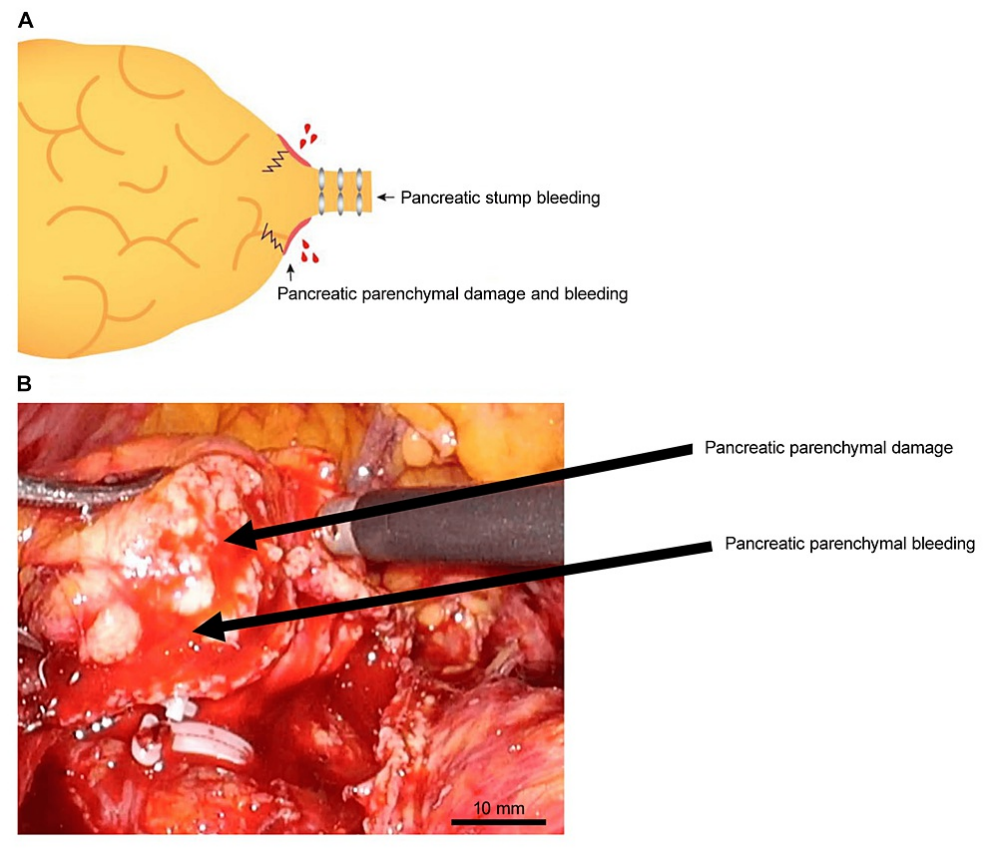
Pancreatic parenchyma damage and bleeding due to stapler closure.

CT and intraoperative videos were evaluated by two surgeons (HY and HI). Pancreatic thickness was measured on pre- and postoperative CT after confirming the resection line. Measurements were recorded based on the preoperative CT coronal section. The surgeon determined the hardness of the pancreas, which has no clear criterion. Intraoperative videos of pancreatic dissection were retrospectively reviewed, and pancreatic parenchymal damage, pancreatic parenchymal bleeding, and pancreatic stump bleeding were evaluated (Figure 1). Pancreatic stump bleeding included bleeding from the pancreatic parenchyma and stapler.

POPF and definition and grading of complications

POPF diagnosis was made using the International Study Group of Pancreatic Surgery (ISGPS) criteria [20]. Cases without clinical impact, particularly those whose amylase levels in the drain fluid on postoperative day 3 were >3 times the upper limit of the normal serum amylase level, were diagnosed with biochemical leaks. Pancreatic fistula cases requiring a change in drain management (e.g., persistent drainage for 3 weeks, percutaneous or endoscopic drainage, vascular embolization for bleeding, or signs of infection without organ failure) were classified as grade B. Pancreatic fistula cases leading to significant changes in clinical management (e.g., reoperation, organ failure, or death) were classified as grade C.

Postoperative complications were classified according to the Clavien-Dindo classification [21]: ≥grade 3 complications were defined as severe complications.

Statistical analyses

All statistical analyses were performed using Statistical Package for the Social Sciences (version 26, IBM, Armonk, USA). Summary statistical data were obtained using established methods and were presented as percentages or medians. Continuous variables were presented as medians and ranges and compared using the Mann-Whitney U test. Pearson’s chi-squared and Fisher’s exact tests were used to analyze nominal variables. Multivariate analysis was performed using binary logistic regression for variables showing significant differences in univariate analysis. A p-value of <.05 was considered to indicate statistical significance.

Patient and public involvement statement

This study aims to investigate past events by gathering information on patient’s post-hospital discharge. The research questions and outcome measures were crafted based on the participants’ priorities, experiences, and preferences, guided by the Tokai University Ethics Committee. The data collection process was retrospective, utilizing only participant information. Participants were not required to assess the intervention’s burden or the time commitment for research participation. The dissemination of the research findings was carried out in accordance with ethical guidelines through an ethics committee

## Results

Patient characteristics

The characteristics of the 54 patients included in this study. The median patient age was 71 years, with a male-to-female ratio of 13:14 and a median BMI of 22.3 kg/m2. The median pancreatic thickness, as measured via CT coronal section, was 20.0 (6.8-35.4) mm.

Regarding surgical factors, the median operative time was 183.5 min, and the median blood loss was 100 mL. Regarding pancreatic texture, 79.6% of cases had a soft pancreas, whereas 20.4% had a hard pancreas. The incidence of pancreatic stump bleeding, pancreatic parenchymal bleeding, and pancreatic parenchymal damage was 61.1%, 22.2%, and 29.6%, respectively.

Regarding postoperative factors, ISGPS grade B/C pancreatic fistula was observed in 12 patients (22.2%); however, no grade C pancreatic fistulas were observed. Morbidity other than pancreatic fistula was observed in two patients (3.7%) with a Clavien-Dindo grade of ≥IIIa. One patient required reoperation owing to gastrointestinal perforation, and the other patient developed pneumonia. The median length of postoperative hospital stay was 8 (4-156) days. Six patients (11.1%) underwent readmission within 30 days, of whom five patients were readmitted because of intraperitoneal abscess due to pancreatic fistula and one patient was readmitted because of dehydration. The median tumor size was 33.0 mm. Pancreatic ductal adenocarcinoma (PDAC) accounted for 25 cases (46.2%), and the remaining cases were due to other diseases (53.8%) (Table 1).

**Table 1:** Patient characteristics (n = 54). Alb: albumin; HbA1c: glycated hemoglobin; AMY: amylase; PDAC: pancreatic ductal adenocarcinoma; IPMN: intraductal papillary mucinous neoplasm; NET: neuroendocrine tumor; MCN: mucinous cystic neoplasm of the pancreas; SCN: suprachiasmatic nucleus; SPN: solitary pulmonary nodule; ISGPS: International Study Group of Pancreatic Surgery

Variables	Median (range) or No. (%)
Preoperative factor	
Age	71 (30–86)
Sex	
Male	26 (48.1%)
Female	28 (51.9%)
Body mass index (kg/m^2^)	22.3 (12.4–48.7)
Diabetes	29 (53.7%)
Dialysis	3 (5.6%)
Alb (g/dL)	4.0 (2.4–4.7)
HbA1c (%)	7.15 (5.0–10.8)
Pancreatic thickness (mm)	20.0 (6.8–35.4)
Operative factors	
Operative time (min)	183.5 (111–352)
Blood loss (mL)	100 (0–500)
Pancreatic texture (soft:hard)	43 (79.6%):11 (20.4%)
Pancreatic stump bleeding	33 (61.1%)
Pancreatic parenchymal bleeding	12 (22.2%)
Pancreatic parenchymal damage	16 (29.6%)
Postoperative and pathological factors	
Pancreatic fistula ISGPS grade B/C	12 (22.2%)
AMY level on postoperative day 1 (IU/L)	1,813 (62–14,497)
AMY level on postoperative day 3 (IU/L)	188 (17–2,119)
Morbidity (Clavien–Dindo) ≥ Ⅲa	2 (3.7%)
In-hospital stay (days)	8 (4–156)
Readmission (within 30 days)	6 (11.1%)
Tumor size (mm)	33.0 (11–105)
Histological factors	
PDAC	25 (46.2%)
IPMN	10 (18.5%)
NET	6 (11.1%)
MCN	2 (3.7%)
SCN	5 (9.3%)
SPN	1 (1.9%)
Others	5 (9.3%)

Pancreatic and nonpancreatic fistula groups and univariate analysis of preoperative factors

No significant differences in nutritional status, such as BMI, pancreatic cancer, or albumin levels, were observed between the pancreatic and nonpancreatic fistula groups. Pancreatic thickness in the pancreatic and nonpancreatic fistula groups, as measured via CT coronal section, was 21.8 and 20.0 mm, respectively, with no significant differences between the two groups (Table 2).

**Table 2:** Univariate analysis of preoperative factors for patients in the pancreatic and nonpancreatic fistula group. PDAC: pancreatic ductal adenocarcinoma; Alb: albumin; HbA1c: glycated hemoglobin.

Variables	Pancreatic fistula group n = 12	Nonpancreatic fistula group n = 42	p-value
Age (median, range)	67 (38–82)	72 (20–86)	0.360
Male:Female	7:5	19:23	0.318
Body mass index (kg/m^2^; median)	24.0 (18.7–48.7)	21.9 (12.4–32.5)	0.105
Histology PDAC	3 (25%)	20 (47.6%)	0.143
Diabetes	7 (58.3%)	22 (52.4%)	0.487
Dialysis	2 (16.7%)	1 (2.4%)	0.121
Alb level (g/dL; median)	4.4 (2.4–4.7)	4.0 (3.0–4.5)	0.237
HbA1c level (%; median)	6.85 (5.0–8.0)	7.6 (5.3–10.8)	0.077
Pancreatic thickness (mm; median)	21.8 (10.1–30.1)	20.0 (6.8–35.4)	0.492

Univariate and multivariate analyses of operative and postoperative factors

No significant differences in operative time and blood loss were noted between the two groups. As expected, the pancreatic fistula group had a significantly higher incidence of complications with Clavien-Dindo grade of ≥IIIa, longer hospital stays, and a higher number of readmissions than the nonpancreatic fistula group. Furthermore, the pancreatic fistula group had a significantly higher number of cases of hard pancreas (p =.009), pancreatic parenchymal bleeding (p =.002), and pancreatic parenchymal damage (p <.001). However, no significant differences were observed in terms of pancreatic stump bleeding (p =.561), which was considered unrelated to pancreatic fistulas (Table 3). Multivariate analysis revealed that pancreatic parenchymal damage was an independent factor associated with pancreatic fistula (hazard ratio, 81.4 (8.5-772.3), p <.001; Table 4).

**Table 3:** Univariate analyses of operative and postoperative factors.

Variables	Pancreatic fistula group n = 12	Nonpancreatic fistula group n = 42	p-value
Operative time (minutes; median)	187 (144–276)	182 (111–352)	0.917
Blood loss (mL; median)	135 (10–500)	91.5 (5–430)	0.208
Morbidity (Clavien–Dindo grade of ≥Ⅲa)	2 (16.7%)	0 (0%)	0.046
Hospital stay (day; median)	15.5 (6–165)	7 (4–16)	<0.001
Readmission	5 (41.6%)	1 (2.4%)	<0.001
Pancreatic texture (soft:hard)	6:6	37.5	0.009
Pancreatic stump bleeding	8 (66.7%)	22 (52.4%)	0.561
Pancreatic parenchymal bleeding	7 (58.3%)	5 (11.9%)	0.002
Pancreatic parenchymal damage	11 (91.6%)	5 (11.9%)	<0.001

**Table 4:** Results of the multivariate analysis. CI: confidence interval.

Variables	Univariate analysis p-value	Multivariate analysis Odds ratio (95% CI)	p-value
Pancreatic texture (soft:hard)	.009	-	-
Pancreatic parenchymal bleeding	.002	-	-
Pancreatic parenchymal damage	< .001	81.4 (8.5–772.3)	< .001

## Discussion

This study retrospectively analyzed factors that may cause POPF based on the magnification effect of laparoscopy, which was used to observe stapler closure of the pancreatic stump during Lap-DP. Currently, the reported incidence of POPF following Lap-DP varies between 5.5% and 35% [9-15]. The lowest incidence reported by Matsumoto et al. [15], who utilized the slow firing method, was 5/73 cases (5.5%); however, this study excluded three patients with severe pancreatic parenchymal damage due to stapling. Currently, POPF due to automatic staplers occurs in approximately 10%-30% of cases [10-15]. In this study, the incidence of pancreatic fistula was 22.2%.

Previous studies have identified pancreatic stump thickness and bleeding at the pancreatic cut edge as potential causes of pancreatic fistula [13,16-19]. A previous study revealed that pancreatic stump thickness of >14 mm is associated with a higher incidence of pancreatic fistula [12]. Several multicenter RCTs have reported that pancreaticojejunostomy results in decreased rates of grade B/C pancreatic fistula when the pancreatic stump thickness is >12 mm [19]. In the present study, no significant difference in pancreatic stump thickness was noted between the pancreatic and nonpancreatic fistula groups, as measured via coronal CT. This may be due to the small number of cases.

In our study, there were no significant differences in patient background. Pancreatic parenchymal injury due to the pressure of the automatic suturing device was considered an independent risk factor for pancreatic fistula. Video data revealed that the pancreatic parenchymal injury occurred slightly away from the stapler using reinforced staplers, no significant reduction in the size of the pancreatic fistula was observed because of the pressure of the device. Pancreatic stump bleeding occurs if the pancreatic parenchyma is torn. No correlation was observed between pancreatic stump bleeding and pancreatic fistula. Although pancreatic thickness is a factor that makes the pancreatic parenchyma prone to tearing, pancreatic hardness is also important. A hard pancreas is more prone to pancreatic parenchymal tears. However, there are no clear criteria for determining pancreatic hardness, and tactile sensation is not available with laparoscopy. Modalities for measuring pancreatic hardness should be investigated. In this study, significant differences in pancreatic thickness were not observed; this may be because pancreatic thickness cannot be measured via coronal CT alone. As the pancreas is a solid organ, it may need to be evaluated based on volume.

To date, using Lap-DP, pancreatic fistula prevention has been reported in almost of cases through the closure of the pancreatic transection edge with an automatic stapler [9-15]. RCTs based on the application of PGA mesh sheets over the pancreatic transection edge have been conducted, demonstrating the effectiveness of this technique [14]. PGA mesh sheet may be useful for patients with pancreatic parenchymal damage. However, even in the PGA group, the incidence of pancreatic fistula was 11.4%.

This was a single-center retrospective study. To obtain higher-quality evidence, prospective trials involving multiple centers are warranted. This study may contribute to the reduction of POPF cases using Lap-DP.

## Conclusions

We used the magnification effect of the laparoscope to observe the pancreatic fragment of stapler closure by Lap-DP, which suggests that one of the causes of pancreatic fistula may be pancreatic parenchymal damage.
